# Anaemia is an essential complication of ANCA-associated renal vasculitis: a single center cohort study

**DOI:** 10.1186/s12882-017-0754-8

**Published:** 2017-11-25

**Authors:** Tetsuya Kawamura, Joichi Usui, Shuzo Kaneko, Ryoya Tsunoda, Eri Imai, Hirayasu Kai, Naoki Morito, Chie Saito, Michio Nagata, Kunihiro Yamagata

**Affiliations:** 1 Department of Nephrology, Faculty of Medicine, University of Tsukuba, 1-1-1 Tennodai, Tsukuba, Ibaraki 305-8575 Japan; 2 Department of Pathology, Faculty of Medicine, University of Tsukuba, Tsukuba, Ibaraki Japan

**Keywords:** Anaemia of chronic disease, Renal anaemia, ANCA-associated renal vasculitis

## Abstract

**Background:**

Anaemia is a common complication of patients with antineutrophil cytoplasmic antibody (ANCA)-associated renal vasculitis. Nevertheless, the cause and degree of such cases of anaemia have not been elucidated in detail. We aimed to investigate the prevalence, cause, pathogenesis of anaemia and the impact of anaemia on prognosis in patients with ANCA-associated renal vasculitis.

**Methods:**

We identified 45 patients with ANCA-associated renal vasculitis that were clinically and/or histologically diagnosed and treated from 2003 to 2014 at University of Tsukuba Hospital. The relationships between anaemia and various clinicopathological findings were evaluated.

**Results:**

At the time of diagnosis of ANCA-associated renal vasculitis, all patients showed anaemia, with a mean haemoglobin level of 7.5 ± 1.3 g/dL. Renal anaemia was diagnosed in 92% of patients, anaemia of chronic disease (ACD) in 56%, and anaemia due to hemorrhage in 20%. Next, the patients were divided into two groups according to anaemia severity: minimum haemoglobin (min Hb) < 7.5 (*n* = 24) and min Hb ≥ 7.5 (*n* = 21). A comparison of baseline characteristics showed that serum albumin, maximum serum creatinine, minimum estimated glomerular filtration rate (eGFR), serum cystatin C, and the area of tubulointerstitial damage were significantly different between the haemoglobin groups (*p* <  0.05). No significant intergroup differences were observed in iron-related or inflammation-related data. With regard to the relationship between anaemia severity and prognosis, patients in the min Hb < 7.5 group tended to have a lower eGFR. Anaemia severity was associated with markedly lower survival (Log-rank test, *p* = 0.03).

**Conclusions:**

In this cohort of patients with ANCA-associated renal vasculitis, all subjects exhibited anaemia. In regard to the cause and pathogenesis, the most prevalent form of anaemia was renal anaemia, not ACD, and a potential reason for the high prevalence of anaemia in our cohort may have been the interaction between renal anaemia and ACD. Moreover, anaemia severity was significantly associated with the degree of renal dysfunction and life prognosis.

## Background

Antineutrophil cytoplasmic antibody (ANCA)-associated renal vasculitis is a systemic autoimmune disease characterized by pauci-immune-type necrotizing small-vessel vasculitis; vessels in the kidney, skin, respiratory tract, gastrointestinal tract, and peripheral nerves are often involved. Patients with ANCA-associated renal vasculitis may present with a variety of clinical manifestations, such as fatigue, fever, and weight loss [1]. Although routine laboratory tests are generally nonspecific for ANCA-associated renal vasculitis, common laboratory findings in ANCA-associated renal vasculitis include leukocytosis, thrombocytosis, normochromic and normocytic anaemia, and the elevation of acute-phase inflammatory proteins. For example, a previous study reported that anaemia was seen in 73% of patients with granulomatosis with polyangiitis (GPA) and the mean haemoglobin was 11.1 g/dL (range, 5.0–15.1 g/dL) before treatment initiation [2]. In another study, 14/36 (39%) of GPA patients with remission status presented with anaemia, and the mean haemoglobin was 13.0 ± 2.1 g/dl [3]. Thus, anaemia is known to be a common complication of patients with ANCA-associated renal vasculitis.

Anaemia has also been reported as a complication in other autoimmune inflammatory diseases, such as rheumatoid arthritis [4], inflammatory bowel disease [5, 6] and systemic lupus erythematosus (SLE) [7]. Anaemia that occurs as a complication in these diseases is generally known as anaemia of chronic disease (ACD; also called anaemia of inflammation) [8]. The mechanisms of ACD are thought to be hepcidin-induced changes in iron metabolism, inadequate response of erythropoiesis, and shortening of the erythrocyte lifespan. A number of serious conditions are associated with ACD, including infections, malignancies, autoimmune diseases, chronic rejection after transplantation, and chronic kidney disease (CKD). ACD is automatically presumed to be complicated with ANCA-associated renal vasculitis, and the causes of anaemia with ANCA-associated renal vasculitis are expected to be multifactorial. These causes can include renal dysfunction, alveolar haemorrhage, malnutrition, the use of immunosuppressive drugs, frequent in-hospital phlebotomies and iron deficiency. However, the associations among these factors and the severity of anaemia in patients with ANCA-associated renal vasculitis have not been elucidated in detail. Moreover, almost all previous studies of anaemia in association with other autoimmune inflammatory diseases excluded patients with renal dysfunction. Therefore, the pathogenesis and severity of anaemia in patients with ANCA-associated renal vasculitis may differ from those of anaemia in patients with other autoimmune inflammatory diseases.

In general, anaemia in elderly patients is closely associated with various poor outcomes such as hospitalization and mortality [9]. In patients with heart failure, anaemia is associated with increased mortality [10]. Additionally, in patients with CKD, several studies have demonstrated that anaemia is closely related to mortality and the progression of renal failure [11, 12]. Therefore, the combination of severe anaemia and ANCA-associated renal vasculitis may lead to poor renal prognosis and shortened lifespan.

In the present study, we aimed to investigate the prevalence and pathogenesis of anaemia in patients with ANCA-associated renal vasculitis. We also evaluated the pathological findings and the impact of anaemia on the renal and life prognoses of the patients.

## Methods

### Study population

We identified 45 patients with ANCA-associated renal vasculitis who had been clinically and/or histologically diagnosed and treated from 2003 to 2014 at University of Tsukuba Hospital. We recorded the demographic, clinical and pathological features of these cases as well as the treatments, patient survival rates and renal outcomes. This research complied with the Declaration of Helsinki and was approved by the Ethics Committee of University of Tsukuba Hospital (H20–273, H24–169 and H26–175). All participants provided informed consent to participate in the study as required by the Institutional Review Board (H20–273). An announcement of this study was simultaneously posted at the outpatient clinic of our institute.

### Definition of anaemia

According to the World Health Organization, anaemia is defined by haemoglobin levels lower than 13.0 g/dL for men and 12.0 g/dL for women [13]. Renal anaemia is defined as the presence of anaemia in association with a relatively low erythropoietin concentration (< 50 mIU/mL) or with a low estimated glomerular filtration rate (eGFR) (< 30 min/mL/1.73 m^2^) but without an increase in the reticulocyte count (< 10 × 10^4^ /μL) [14, 15]. The definition of ACD requires a low transferrin saturation (< 16%) with a normal or increased serum ferritin concentration (>100 ng/mL). Iron-deficiency anaemia is characterized by the presence of anaemia in association with low serum ferritin (< 10 ng/mL for females, < 15 ng/mL for males) or with a transferrin saturation of less than 16% together with a serum ferritin level of less than 30 ng/mL. The combination of iron-deficiency anaemia and ACD is characterized by a transferrin saturation of less than 16% and a serum ferritin between 30 and 100 ng/mL inclusive [6, 8]. Moreover, in the present study, we evaluated the serum hepcidin-25 concentration to use as a reference value for ACD. The serum hepcidin-25 concentration was measured using a commercially available enzyme-linked immunosorbent assay (ELISA) kit (product No. S-1337; Peninsula Laboratories, Bachem, CA). This ELISA system was used in a number of previous studies [16, 17]. If patients had additional cytopenia (white blood cell count < 4000/μL, platelet count < 10 × 10^4^/μL) and/or abnormal white blood cell differentiation, haematological abnormalities were suspected and we added a haematological examination, including bone marrow aspiration. Haemolysis was diagnosed if the lactate dehydrogenase level was greater than 500 IU/L without the elevation of another liver enzyme or if the haptoglobin level was less than 10 mg/dL. Vitamin B12 deficiency was diagnosed if the serum vitamin B12 concentration was less than 200 pg/mL, and folate deficiency was diagnosed if the serum folate level was less than 3.6 ng/mL.

### Pathologic analysis

Renal biopsy specimens were routinely assessed by light microscopy, immunofluorescence, and electron microscopy. An expert renal pathologist made the diagnoses according to the international histological classification [18]. The area of tubulointerstitial damage was semi-quantitatively evaluated in renal cortical tissue [19–21].

### Statistical analysis

The laboratory examination results were summarized in the form of percentages, means ± standard deviations (SD), or medians with ranges. To perform statistical comparisons, we used the chi-squared test for categorical variables and Student’s *t*-test or the Mann-Whitney U test for continuous variables. The cumulative survival and renal survival were estimated using the Kaplan-Meier method and compared among the groups using the log-rank test. A *p* value < 0.05 was considered statistically significant in all analyses. The IBM SPSS software package, ver. 22, was used.

## Results

### Patient characteristics at diagnosis

The patient characteristics at diagnosis are shown in Table 1. Of the 45 patients, 20 (44%) were men. The mean age at diagnosis was 71 ± 7.8 years. Two patients already had renal dysfunction and 8 patients showed mild anaemia (haemoglobin 10.1–12.9 g/dL) before the disease onset. Eighteen patients had a history of hypertension, 2 had a history of diabetes, 3 had a history of cardiovascular disease, 4 had a history of malignancy, and 1 patient had a history of rheumatoid arthritis. In this cohort, the median follow-up duration was 42 months (range, 0–123 months). All patients were myeloperoxidase-ANCA single-positive. With respect to the vasculitis disease classification, physicians diagnosed microscopic polyangiitis in 43 patients, eosinophilic granulomatosis with polyangiitis in 1 patient, and GPA in 1 patient. The median Birmingham Vasculitis Activity Score was 20. The mean haemoglobin on admission was 9.0 g/dL, and the mean minimum haemoglobin (min Hb) was 7.5 g/dL. The median serum erythropoietin was 15.1 mIU/mL (based on measurement in 25 patients), the median serum ferritin was 322 ng/mL, the median serum c-reactive protein was 6.8 mg/dL, the median serum hepcidin-25 was 71.5 ng/mL (based on measurement in 15 patients) and the median minimum eGFR was 15.3 mL/min/1.73 m^2^ (range, 3.9–54.2 mL/min/1.73 m^2^). All patients had renal involvement, and 33 patients (73%) had undergone renal biopsy. Using the international histological classification [18], we identified 8 patients in the Focal class, 12 in the Crescentic class, 7 in the Mixed class, and 2 in the Sclerotic class. In 4 patients, the renal biopsy specimens were inadequate for pathological diagnosis. The mean area of tubulointerstitial damage was 55%. Twenty-three patients (51%) had pulmonary involvement, including 20 with interstitial pneumonia, 5 with alveolar haemorrhage, and 2 with pulmonary granuloma.

**Table 1:** Clinicopathological findings of patients with ANCA-associated renal vasculitis in each anaemia severity group

Variable	All patients	Min Hb <7.5	Min Hb ≥7.5	*P* Value
Patient number	45	24	21	
Gender male:female	20:25	12:12	8:13	0.42
Age (years)	71 ± 7.8 (47–82)	72 ± 7.0 (54–82)	70 ± 8.7 (47–82)	0.36
Birmingham vasculitis activity score	20 (12–39)	19 (12–36)	20 (12–39)	0.58
Haemoglobin on admission (g/dL)	9.0 ± 1.6	8.1 ± 1.1	10.1 ± 1.3	<0.001
Minimum haemoglobin (g/dL)	7.5 ± 1.3	6.5 ± 0.6	8.7 ± 0.9	<0.001
MCV (fL)	90 ± 6.2	88 ± 6.8	91 ± 5.1	0.18
MCH (pg)	29.5 ± 2.4	29.1 ± 2.6	30.0 ± 2.2	0.21
MCHC (%)	33.0 ± 1.2	32.9 ± 0.9	33.0 ± 1.3	0.76
Reticulocyte count (×10^4^/μL)	4.65 ± 2.50	4.23 ± 2.47	5.09 ± 2.54	0.35
Serum erythropoietin (mIU/mL)	15.1 (5.8–105)	17.5 (5.8–105)	14.5 (11–36.6)	0.43
Transferrin saturation (%)	14.7 (4.2–80.2)	12.8 (4.2–80.2)	17.5 (9.7–54.5)	0.18
Serum ferritin (ng/mL)	322 (46.9–2231)	382 (46.9–1839)	291 (94.9–2231)	0.21
Serum c-reactive protein (mg/dL)	6.8 (0.03–24.5)	7.9 (0.43–24.5)	5.0 (0.03–17.3)	0.09
Serum hepcidin-25 (ng/mL)	71.5 (10.5–463)	74.9 (45.9–463)	37 (10.5–363)	0.28
Serum albumin (g/dL)	2.6 ± 0.8	2.3 ± 0.6	2.9 ± 0.8	0.004
Serum creatinine on admission (mg/dL)	2.5 (1.0–10.8	2.9 (1.0–10.8)	2.3 (1.0–6.3)	0.06
Maximum serum creatinine (mg/dL)	3.2 (1.0–11.7)	4.4 (1.1–11.7)	2.4 (1.0–7.2)	0.01
eGFR on admission (mL/min/1.73 m^2^)	19.5 (4.2–54.2)	13.8 (4.2–44.6)	21.6 (7.0–54.2)	0.09
Minimum eGFR (mL/min/1.73 m^2^)	15.3 (3.9–54.2)	8.9 (3.9–39.2)	19.0 (4.8–54.2)	0.01
Blood urea nitrogen (mg/dL)	34.5 (13–101)	36.9 (13–101)	30.9 (18.3–62)	0.09
Serum cystatin c (mg/L)	2.8 ± 0.9	3.2 ± 0.9	2.2 ± 0.6	0.02
Number of renal biopsy, n (%)	33 (73)	13 (54)	20 (95)	0.00
Area of tubulointerstitial damage (%)	55 ± 21	65 ± 18	48 ± 20	0.048

Values are shown as the numbers of patients, the median (range) or the mean ± SD. *Abbreviations*: *Min Hb* Minimum haemoglobin, *MCV* Mean corpuscular volume, *MCH* Mean corpuscular haemoglobin, *MCHC* Mean corpuscular haemoglobin concentration, *eGFR* Estimated glomerular filtration rate

### Prevalence and pathogenesis of anaemia with ANCA-associated renal vasculitis.

**Fig. 1:**
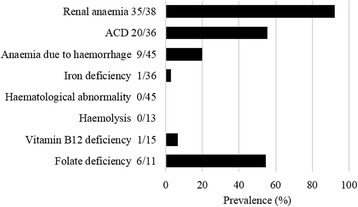
The causes of anaemia in patients with ANCA-associated renal vasculitis. Abbreviation: ACD, anaemia of chronic disease

In this study, we focused on the relationship between ANCA-associated renal vasculitis and anaemia. At the time of the diagnosis of ANCA-associated renal vasculitis, all patients showed anaemia (the haemoglobin on admission was 9.0 ± 1.6 g/dL and the min Hb was 7.5 ± 1.3 g/dL). Age and gender had no significant influence on the severity of anaemia. The causes of anaemia are summarized in Fig. 1. Renal anaemia was diagnosed in 35 of 38 (92%) patients, ACD was diagnosed in 20 of 36 (56%) patients (one patient was diagnosed with iron-deficiency anaemia-associated ACD), and anaemia due to haemorrhage was diagnosed in 9 of 45 (20%) patients. No patients were suspected to exhibit haematological abnormalities or haemolysis. Vitamin B12 deficiency was diagnosed in 1 of 15 (7%) patients, and folate deficiency was diagnosed in 6 of 11 (55%) patients. However, none of the patients exhibited macrocytic anaemia, and none required vitamin B12 or folate supplementation. Therefore, we considered that vitamin B12 and folate deficiency did not greatly affect the anaemia severity.

### Comparison of backgrounds and prognosis according to anaemia severity

Next, we investigated the features of the patients that were influenced by the anaemia severity. The patients were divided into two groups according to their min Hb values: a group with min Hb < 7.5 (*n* = 24) and a group with min Hb ≥ 7.5 (*n* = 21) (Table 1). A comparison of the baseline characteristics showed that serum albumin, maximum serum creatinine, minimum eGFR, serum cystatin C, and the area of tubulointerstitial damage were significantly different between the two groups. No significant intergroup differences were observed in iron-related data such as transferrin saturation and serum ferritin, or in inflammation-related data such as serum ferritin, serum c-reactive protein, and serum hepcidin-25.

Next, a comparison of the treatments and prognoses is shown in Table 2. Not surprisingly, the transfusion of red blood cells and the use of erythropoiesis-stimulating agents (ESAs) were more frequent in the min Hb < 7.5 group than the min Hb ≥ 7.5 group. Of the treatments used for ANCA-associated renal vasculitis, plasma exchange therapy was chosen significantly more often in the min Hb < 7.5 group than in the min Hb ≥ 7.5 group. The indication for plasma exchange therapy in these 6 patients was alveolar haemorrhage in 3 patients and severe renal involvement in 3 patients. Three out of 5 patients with alveolar haemorrhage were treated with plasma exchange. One of the remaining 2 patients was not treated with plasma exchange because the alveolar haemorrhage was considered to be resolved. Although we reviewed the relevant medical records, we could not determine why the other patient was treated without plasma exchange. By contrast, the rates of treatment with steroid pulse therapy and of treatment using cyclophosphamide at 4 and 12 weeks after treatment initiation were similar between the two groups.

**Table 2:** Comparison of treatments and prognoses

	All patients	Min Hb <7.5	Min Hb ≥7.5	*P* Value
Treatment				
Blood transfusion of RBC, *n* (%)	13 (29)	12 (50)	1 (5)	0.001
Use of ESAs, *n* (%)	20 (44)	16 (67)	4 (19)	0.001
Plasma exchange therapy, *n* (%)	6 (13)	6 (25)	0 (0)	0.01
Steroid pulse therapy, *n* (%)	20 (44)	13 (54)	7 (33)	0.16
Use of CY within 4 weeks after treatment initiation, *n* (%)	1 (2)	1 (4)	0 (0)	0.33
Use of CY within 12 weeks after treatment initiation, *n* (%)	14 (35)	9 (47)	5 (24)	0.12
Prognosis				
Duration of hospitalization (days)	70 ± 26	75 ± 28	65 ± 23	0.23
Number of deaths during the first hospitalization, *n* (%)	4 (9)	4 (17)	0 (0)	0.05
Number of deaths at last follow-up, *n* (%)	11 (24)	8 (33)	3(14)	0.14
Number of patients with end-stage kidney disease at last follow-up, *n* (%)	2 (4)	2 (8)	0 (0)	0.18

Values are shown as the numbers of patients or the mean ± SD. *Abbreviations*: *Min Hb* Minimum haemoglobin, *RBC* Red blood cell, *ESAs* Erythropoiesis-stimulating agents, *CY* Cyclophosphamide

**Fig. 2:**
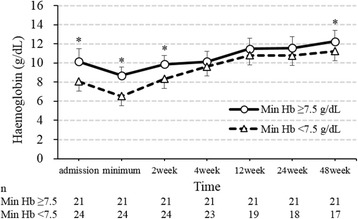
Temporal changes of haemoglobin in each anaemia severity group. Values are shown as the mean ± SD. *p < 0.05. Abbreviation: Min Hb, minimum haemoglobin

**Fig. 3:**
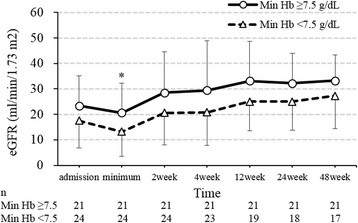
Temporal changes of eGFR in each anaemia severity group. Values are shown as the mean ± SD. *p < 0.05. Abbreviations: Min Hb, minimum haemoglobin; eGFR, estimated glomerular filtration rate

**Fig. 4:**
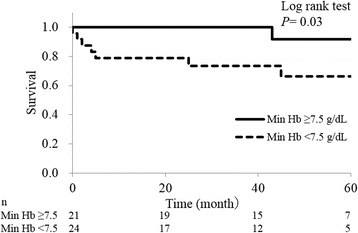
Comparison of the survival rate in each anaemia severity group. Abbreviation: Min Hb, minimum haemoglobin

Finally, we examined the relationship between the severity of anaemia and prognosis (Table 2). There was no significant difference in the duration of hospitalization. However, all 4 patients who died during the first hospitalization were in the min Hb < 7.5 group. Over the course of the follow-up, 11 of 45 (24%) patients died, and 2 of 45 (4%) patients developed end-stage kidney disease. Figures 2 and 3 show the temporal changes in the haemoglobin concentration and eGFR levels. The haemoglobin concentration showed significant differences between the groups upon admission, at the time of min Hb, after 2 weeks, and after 48 weeks. There is a possibility that severe anaemia at diagnosis was prolonged for at least 1 year after the diagnosis. On the other hand, eGFR showed significant differences between the groups at the time of minimum eGFR, and the min Hb < 7.5 group tended to have a lower eGFR. As shown in the survival curve estimated using the Kaplan-Meier method (Fig. 4), increased anaemia severity was associated with a markedly lower survival rate.

## Discussion

In the present study, we focused on the physiological impact of anaemia in cases of ANCA-associated renal vasculitis. All ANCA-associated renal vasculitis patients in this cohort were found to have anaemia, and the causes of anaemia in our patients were expected to be multifactorial, with renal anaemia being the predominant type. Also, the severity of anaemia was closely associated with renal function, but not with the iron-related or inflammation-related findings. Moreover, anaemia severity was a factor affecting the renal and life prognoses of ANCA-associated renal vasculitis patients.

Before discussing our findings, we should first consider the previous studies on anaemia in patients with other autoimmune inflammatory diseases. The previous studies reported that the prevalence of anaemia was 33–59% in patients with rheumatoid arthritis [4], 20–68% in those with inflammatory bowel disease [5] and 38% in those with SLE [7]. According to these studies, the causes of anaemia were multifactorial, and the main cause was ACD. In comparison, we found that anaemia was more frequent in patients with ANCA-associated renal vasculitis, and the causes of anaemia with ANCA-associated renal vasculitis were multifactorial, just as for anaemia with other autoimmune inflammatory diseases. However, the most prevalent cause was renal anaemia, not ACD. More than 90% of our patients had renal anaemia, while approximately half of the patients had ACD. A possible reason for the high prevalence of anaemia in our patients with ANCA-associated renal vasculitis may have been the interaction between renal anaemia and ACD. As described above, renal dysfunction is an important cause of ACD [8]. By contrast, inflammation is an essential cause of renal anaemia [22]. Both renal anaemia and ACD are mediated through the effects of inflammatory cytokines such as interleukin (IL)-1, IL-6, and tumour necrosis factor-alpha. Therefore, in this study, we suspected that hepcidin played a key role in the interaction between renal anaemia and ACD. Hepcidin is an acute-phase protein, which is mainly produced in the liver and secreted into the circulation. Hepcidin is the main regulator of iron metabolism, and its production is regulated by changes in the body’s iron stores, inflammation, erythropoietic activity, and hypoxia [23]. It has been reported that the hepcidin level is increased in CKD patients, possibly due to increased production and/or reduced renal clearance [24, 25]. Hepcidin is also an acute-phase reactant induced by inflammation. We therefore expected the hepcidin concentration to be increased in our patients with ANCA-associated renal vasculitis due to both renal dysfunction and inflammation. However, in this cohort, the hepcidin-25 concentration was not significantly related to anaemia severity, renal dysfunction, or other inflammatory findings. In patients with ANCA-associated renal vasculitis, there may be a more complex relationship among various factors that influence hepcidin levels, including iron, inflammation, reduced renal clearance, and anaemia severity [26]. Additionally, the measurement of the serum hepcidin-25 concentration was performed in only a limited number of patients.

Next, we addressed the reason for the high prevalence of renal anaemia and the influence of renal anaemia on the severity of anaemia in our patients with ANCA-associated renal vasculitis. McClellan et al. [15] reported that the prevalence of anaemia increased as the level of kidney function decreased. For example, the percentage of patients with haemoglobin ≤12 g/dL increased from 26.7% to 75.5% as the glomerular filtration rate decreased from ≥ 60 mL/min/1.73 m^2^ to < 15 mL/min/1.73 m^2^. In our cohort, all patients had renal involvement, and the median eGFR was 15.3 mL/min/1.73 m^2^. Therefore, advanced renal dysfunction with ANCA-associated renal vasculitis corresponded to a high prevalence of renal anaemia and anaemia severity. We also performed a review of the literature to examine how renal anaemia affects anaemia severity in patients with ANCA-associated vasculitis (Table 3). Several previous studies have reported an association between renal dysfunction and anaemia severity. However, anaemia severity was relatively mild in the studies that included patients without renal involvement—that is, in patients with preserved renal function [2, 3, 27]. On the other hand, anaemia severity was relatively advanced in the ANCA-associated renal vasculitis cohorts [28–30]. Moreover, there may be a tendency for the severity of anaemia to progress as the level of kidney function decreases. As a result, we can conclude that the causes of anaemia with ANCA-associated renal vasculitis are multifactorial, and that while renal anaemia is easily missed, it is the most frequent and influential cause of anaemia in patients with ANCA-associated renal vasculitis.

**Table 3:** Association of renal dysfunction and anaemia severity in patients with ANCA-associated vasculitis

Author	Disorder	Patient number	Age (years)	Patients with renal involvement (%)	Serum creatinine (mg/dL)	Haemoglobin (g/dL)
Riegersperger et al. [3]	GPA (remission status)	36	58 ± 15	42% had pauci-immune crescentic glomerulonephritis	(eGFR 40.6 ± 21.4 ml/min/1.73 m^2^)	13.0 ± 2.1
Hoffman et al. [2]	GPA	158	41 (range, 9–78)	77	not available	11.1 (range, 5.0–15.1)
Flossmann et al. [27]	ANCA-associated vasculitis	535	61 (49–69)	not available	2.3 (1.1–5.6)	9.8 (8.6–11.5)
Crnogorac et al. [28]	ANCA-associated vasculitis	81	61 (50–68)	100	3.6 (2.3–6.0)	9.8 (8.8–11.7)
Andreiana et al. [29]	ANCA-associated vasculitis	75	60 (53–68)	100	5.0 (3.4–7.9)	8.5 (7.5–9.8)
Pu et al. [30]	ANCA-associated vasculitis	123	62 ± 12	100	5.0 ± 3.8	8.3 ± 2.1
The present study	ANCA-associated renal vasculitis	45	71 ± 7.8	100	3.2 (range, 1.0–11.7)	7.5 ± 1.3

Values are shown as the median (interquartile range) or mean ± SD if not otherwise specified. *Abbreviations*: *GPA* Granulomatosis with polyangiitis, *ANCA* Antineutrophil cytoplasmic antibody, *eGFR* Estimated glomerular filtration rate

Next, we investigated the mechanism of renal anaemia in ANCA-associated renal vasculitis patients. Our study of the histopathological findings demonstrated that renal interstitial damage is associated with anaemia severity. Some recent studies investigated the association between anaemia and biopsy-proven interstitial lesions of diabetic nephropathy [19], or between anaemia and post-transplant nephropathy [20]. Based on the findings of these previous studies and our present investigation, it is possible to propose several mechanisms by which interstitial damage could influence anaemia. One such mechanism is a reduction in the production of erythropoietin resulting from a decrease in erythropoietin-producing cells due to interstitial damage. Recent basic science studies have suggested that renal interstitial fibroblasts produce erythropoietin in response to hypoxia or anaemia [31]. Therefore, the progression of interstitial damage could lead to the development of anaemia, resulting in erythropoietin deficiency. However, in this study, there was no significant relationship between the area of interstitial damage and the serum erythropoietin concentration. Nonetheless, this discrepancy may be attributable to study limitations. That is, we measured the serum erythropoietin concentration only in a limited number of patients. In addition, we could not perform renal biopsies in all patients.

Whether anaemia with ANCA-associated renal vasculitis influences the renal and life prognoses must also be addressed. As described above, several studies have demonstrated that anaemia is a risk factor for shortened lifespan and/or increased renal dysfunction [11, 12]. Similarly, the present data demonstrated that anaemia severity was associated with life prognosis in our patients with ANCA-associated renal vasculitis. On the other hand, eGFR was also significantly different between the groups at the time of minimum eGFR, and the lower haemoglobin group tended to have a lower eGFR. Therefore, our results underscore that the severity of anaemia has an impact on renal and life prognoses. In general, the administration of ESAs is beneficial in patients with renal anaemia [32, 33], and the use of ESAs may be a treatment option for patients with ACD [8]. Unfortunately, although renal anaemia was diagnosed in 35 patients in this cohort, ESAs were used in only 16 of the 35 (46%). There is a possibility that the treatment for anaemia itself would lead to an improvement in the prognosis of patients with ANCA-associated renal vasculitis. In the future, therefore, it will be important to investigate the efficacy of earlier treatment initiation for anaemia, the optimal timing of anaemia intervention and the effectiveness of ESAs therapy.

Finally, our study has several limitations. First, this study was a retrospective investigation of a small cohort at a single centre, and our results therefore cannot be generalized without further investigation. Second, when our patients with ANCA-associated renal vasculitis had both renal anaemia and ACD, it was difficult to clearly distinguish between them. Third, as described above, we could not analyse patients with ANCA-associated vasculitis without renal involvement. Despite these limitations, the present study provided the first investigation of the pathogenesis of anaemia with ANCA-associated renal vasculitis and the impact of anaemia on prognosis. It is hoped that our findings will inspire additional research in a larger cohort.

## Conclusions

We demonstrated a high prevalence of anaemia in a cohort of patients with ANCA-associated renal vasculitis. A possible reason for the high prevalence of anaemia in these patients was the interaction between renal anaemia and ACD. The severity of anaemia was associated with the degree of renal dysfunction and life prognosis.

## Abbreviations

ACD: Anaemia of chronic disease; ANCA: Antineutrophil cytoplasmic antibody; CKD Chronic kidney disease; eGFR: Estimated glomerular filtration rate
ELISA: Enzyme-linked immunosorbent assay
ESAs: Erythropoiesis-stimulating agents
GPA: Granulomatosis with polyangitis
IL: Interleukin
min Hb: Minimum haemoglobin
SD: Standard deviation
SLE: Systemic lupus erythematosus
